# Threefold relationship between ideal humanity and sublime beauty in Chinese art forms

**DOI:** 10.3389/fpsyg.2025.1547607

**Published:** 2025-04-17

**Authors:** Maoyuan Ding, Xiao Tan

**Affiliations:** ^1^Liberal Arts College, Hubei Institute of Fine Arts, Wuhan, China; ^2^Institute of Educational Sciences, Hubei University of Education, Wuhan, China

**Keywords:** sublime beauty, mind, cognition, ideal self, ideal humanity, Chinese art

## Abstract

In Chinese thought, the concept of the “sublime” is closely intertwined with “beauty,” the two concepts merging to form a sublime beauty that seeks the continuous development of universal life amid change. This paper explores the multifaceted relationship between the Chinese artistic mind and sublime beauty through three dimensions of the research model: potential, processes, and goals. In terms of potential, Chinese artistic thought places full trust in the inherent potential of an individual’s ideal humanity, emphasizes the awakening of the sublime beauty within each person through specific art forms, and cultivates different perspectives to understand the connection between the mind and the sublime beauty of our world. In terms of process, Chinese artistic thought is characterized by the focused sincerity of the creative mindset; creative thinking that balances the “degree” of rational expression of emotions and the “*qi*” cultivated through daily spiritual practice; and the cognition of the sublime, which combines the gradual enlightenment gained from daily experiences of embodied cognition with the sudden enlightenment triggered by creative talent. In terms of artistic goals, ideal humanity in pursuit of sublime beauty is divided into three states: the “self state” that manifests the style of the individual subject, the “no-self state” that transcends the limitations of the self, and the “empty state” that surpasses even the cognitive notion of “transcending the self.” The threefold relationship between ideal humanity and sublime beauty in Chinese art can provide contemporary psychology with research model and unique perspectives on being aware of one’s self-potential, understanding their emotions and other aspects of the mind, and constructing multiple value goals.

## 1 Chinese concepts of sublime beauty and ideal humanity

### 1.1 Introduction: sublime beauty in the context of Chinese culture

As an important interdisciplinary concept, the “sublime” encompasses experiences of self-awareness across cultures. However, a critical question arises: how can we explore the potential, processes, and goals common to human psychology by examining the relationship between the Chinese concept of the sublime and the mind? From Pseudo-Longinus onward, the sublime has been understood as a force that elevates the soul, filling one with pride, satisfaction, and flaunted delight (Longinus, 1995, p. 79). Burke (1990, p. 36) argued that the sublime originates from human fear or other strong emotions, whereas Kant (2000, p. 134) claimed that it transcends the limits of the senses. However, from the Western research perspective, especially in the fields of psychology, art and philosophy, the ideas of the sublime in China, and East Asia more broadly, appear to be largely absent. Although some scholars have already affirmed the profundity and metaphysical value of Chinese and East Asian (e.g., Japanese) sublime thought (Brady, 2013, p. 204), yet in-depth discussions on this topic remain sparse. It is precisely because of this cultural gap that Kant (2011, p. 58) perceived the sublime beauty contained within Chinese painting as “grotesque,” (Nelson, 2010) and it is therefore necessary to clarify the ontological concept and spiritual value of Chinese sublime thought (Peng et al., 2006). This also helps to explore how different elicitors in cross-cultural comparisons can inspire common feelings of the sublime, as well as the differences in the expression of sublime experiences (Clewis, 2021).

The following are representative studies on Chinese sublime thought: Pajin (2021), who discussed the sublime experience and rich heritage of classical Chinese poetry. Wong (1980), who explored Chinese sublime beauty from the perspective of Zhuangzi; Lu (2018), who compared the cosmological concepts underlying Zhuangzi and the theory of the sublime; and Zheng (2010), who analyzed the evolution of the sublime in the context of modern Chinese literature. Furthermore, E and Wang (2009) traced the origin and evolution of sublime esthetics in China since the 20th century. Cao and Wang (2017, pp. 94-103) posited that the Western concept of the “sublime” originates from pain, whereas Chinese esthetics favor the “雄渾” (*xiong hun*, grandeur) of optimism and progress.

Overall, there is significant space for further research into the relationship between the mind and psychology. Specifically, research could explore the tangible interactions between the mind and sublime beauty. Additionally, another pertinent question is how to integrate individuals’ potential, processes, and goals of human cognitive sublimity within the aforementioned discussion to construct a universally applicable theoretical model.

To better understand the ontological origins of the “sublime” and “beauty” in China, it is necessary to examine their respective conventions within life philosophy. This helps reveal that beyond the Western academic concepts of “sublime” and “beauty,” China offers its own unique spiritual origins and features of thought regarding these concepts. Etymologically, the term “崇高” (*chong gao*, sublime) in Chinese comes from the *I Ching* (*Classic of Changes*), where it appears in the *Xici* (“*Great Treatise*”) as “崇高莫大乎富貴 (of the sublime and exalted there are none greater than he who is the rich and noble; Kong, 1980, p. 82).” This use of “sublime,” symbolizing the profundity and fullness of earth and the lofty heights of heaven (Li, 2015, pp. 603–604), is the direct object of human cognition of the existence of the sublime. In contrast, the Latin term for “sublime” is “*sublimis*,” composed of “*sub*” (meaning “up to”) and “*limes*” (meaning “lintel”), which emphasizes the idea of elevation to a high place (Shaw, 2017, p. 1). The etymological meanings of “sublime” in both Chinese and Western contexts consider the unity of literal intuition and noble spirit. Some scholars have argued that this term only connotes “nobility” and “reverence,” which lack esthetic value (Chen, 1992, p. 2). This argument holds some merit, but from an ontological perspective, the “sublime” in the *I Ching*, with its universal care for human life, inherently embodies the writer’s aspiration toward an ideal spiritual goal—ideal humanity. A king in a noble position can mobilize the power of the realm to nurture the growth of all things (Kong, 1980, p. 82). In summary, this concern for universal life—this “ultimate good”—is the essence of the Chinese philosophical concept of “*sheng-sheng*” (生生; Kong, 1980, p. 78): understanding and grasping the laws of movement in the world, pursuing innovation amidst change, benefiting every individual, and ultimately achieving the universal survival and ceaseless development of humanity. In comparison, the philosophical sources of Western psychology, such as the ending of “The Republic,” in which Socrates emphasizes the eternity and upwardness of the soul, also pursues the “reward” afterlife and death, and hopes that “we will do well and be happy” in this life or on the thousand-year journey (Cooper, 1997, p. 1223).

In other words, “*sheng-sheng*” represents the sublime goal in which Chinese thought merges individual minds with universal life. “*Sheng”* (生) originates from the life force of the sublime power of heaven and earth. “*Sheng-sheng*” is not a mere repetition of this concept but the continuous forward movement of the entire universe: as the Creator of China, the *Dao* of heaven and earth creates and nurtures all things, including humanity, and propels the organic functioning of the world. Similarly, Kant (2000, pp. 22-24) emphasized that the more universal “true virtue” is, the more sublime it is, with its essence being the pursuit of human beauty. Consequently, regarding the ultimate value of sublime beauty, both Chinese and Western philosophies embody human-centered ideals. The sublime concept of “*sheng-sheng*” is widely expressed in pre-Qin Chinese philosophical texts. Generally, terms such as “*Dao*” (道, Way), “*Wu*” (無, nothing), “*Zaohua*” (造化, the Creator, nature), and “*Tiandi*” (天地, heaven and earth) all emphasize the boundlessness of metaphysical universal love, which benefits humanity. This is consistent with Plato’s *Ideas* and the God of Judaism and Christianity in terms of their sublime ontological status. However, there are systematic differences in the expression of their relationship with humans. Specifically, Confucius praised the virtues and “大 (greatness)” of the ancient sage Emperor Yao (Zhu, 2016, p. 107). Mencius pursued “the beauty of fulfillment (充實之美),” transforming an individual’s inherent sublime potential into self-integrity and manifesting it in concrete forms (Zhu, 2016, p. 378). Laozi viewed the ineffable sublime “*Dao*” as “great,” (Gao, 1996, p. 350) while Zhuangzi considered the beauty of heaven and earth to be a silent “great beauty (大美, *da mei*),” awaiting humanity to become cognizant of its greatness (Guo, 1961, p. 735). These diverse expressions all describe the ontological state, spiritual experience, and practical approach to sublime beauty from different perspectives. Therefore, the concept of the sublime in China inherently encompasses the universal “*sheng-sheng* beauty,” transcending the boundaries of Western and Eastern academic or artistic disciplines. It is widely acknowledged that Chinese thought emphasizes intuitive resonance, which results in a lack of systematic discourse on concepts such as “sublime” and “beauty” within the Chinese tradition. In contrast, the rational framework of Western philosophy provides a scientific foundation for the ongoing analysis of these concepts. In terms of commonality, the concept of the “sublime” is nearly ubiquitous in both Chinese and Western contexts, originating from a positive pursuit of human existence. As Holmqvist and Płuciennik (2002) pointed out, the history of Western philosophy and aesthetics reveals that the “sublime” encompasses numerous theoretical and literary “varieties,” possesses multiple attributes, is linked to various other aesthetic qualities, and has led to the emergence of new interpretations of the “sublime,” such as Benjamin’s notion of “aura.”

In this context, the concept of the “*sheng-sheng*” sublime in China is unified with the concept of beauty. In Chinese, the terms “美” (beauty) and “善” (goodness) are interconnected, emphasizing the interconnected relationship between the true, good, and beautiful aspects of humanity. This aligns with the views of Socrates that beauty and goodness are inherently unified (Marchant and Todd, 1923, pp. 219–220; Cooper, 1997, p. 1038). *Shuowen Jiezi* (說文解字, *Ancient Chinese Etymological Dictionary*) defines “美” as “sweetness; it comprises the characters for ‘羊 (sheep)’ and ‘大 (great)’. The sheep, as one of the six domesticated animals, is served in offerings. Beauty and goodness are synonymous.” (Xu, 1963, p. 78) This explanation has two meanings: the first refers to the fullness of the sheep, which combines sensory beauty in terms of both sight and taste, as well as the inner beauty of its life force; the other refers to the sacrificial offering of such a sheep to deceased ancestors, expressing the reverence and memory of the worshiper. This reverence is manifested and accomplished through a series of rituals by which the individual can also elevate their spiritual cultivation. The unity of “beauty” and “goodness” arises from human cognition and reverence for the sublime, and one’s ability to maintain a sincere state of mind at all times. Modern psychological empirical research has found that there is a complex relationship between awe and the sublime (Arcangeli et al., 2020), and even a large degree of “overlap” (Clewis et al., 2022). Moreover, experiments have revealed that in addition to awe, the sublime also contains inspiring energy, which is defined by “feelings of vitality, joy, energy, oneness, freedom, eternity, and harmony with the universe” (Bethelmy and Corraliza, 2019). On this psychological foundation, the ideal goal of Chinese artistic beauty is to achieve unity with the sublime *Dao*, with artistic beauty flowing naturally from the subject’s ideal humanity, rather than through the deliberate pursuit of such beauty. At a higher level, the unity of the sublime and beauty in many individuals also serves as the premise and goal for humanity as a whole to realize “*sheng-sheng* beauty.” If the sublime is transformed into a psychological “wonder experience,” then the correlation between “awe” and “profound beauty” (Fingerhut and Prinz, 2018) would confirm the identity of the sublime and beauty.

Thus, the homogenous Chinese philosophical concept of sublime beauty directs toward individual and universal “*sheng-sheng* beauty.” For a long time, Chinese discussions of the sublime have focused primarily on “describing the state” (Gao, 2020) rather than developing a concrete concept corresponding to beauty. However, from the perspective of the ontology of life, the “sublime” and “beauty” are not opposing forces. Kant (2011, p. 18) noted that while the sense of the sublime differs from that of beauty, the sublime cannot exist without beauty. Similarly, the Confucian expression of “refined in both literary and material qualities” (文質彬彬; Zhu, 2016, p. 89) indicates that the sense of the sublime arises from the potential for human goodness, which corresponds to “material qualities” (質, *zhi*), and the sense of beauty arises from the elegance of certain forms of art, which can be summarized as “literary qualities” (文, *wen*). Traditional Chinese ideals regarding the spiritual state affirm the shared value of “literary qualities” and “material qualities” in the process of self-cultivation, which can be described in other words as pursuing the unity between the sense of the sublime and that of beauty.

### 1.2 Research model: ideal humanity and potential, process, and goal

How then should we explore the interactive relationship between sublime beauty in Chinese thought and the individual’s own mind? Drawing upon the traditional understanding of Chinese spiritual and sublime beauty, the author introduces the concept of “ideal humanity” as a model to analyze the specific relationship between the two. The definition of “ideal humanity” refers to a state of existence in which individuals freely realize the beauty and value of their humanity. This state does not cease upon the achievement of a goal; rather, it is continually updated, inclusive, and open. The scientific and theoretical psychological foundation of this concept primarily derives from Maslow’s notions of “fully human” or “full humanness.” He posits that this concept can be both descriptive and quantitative, as well as normative, serving to describe “what you would like to be, or what you think people should be like” (Maslow, 1971, pp. 27–28). Examples of this concept arise from common self-reflection and expectations in areas such as learning, job searching, and life more broadly: How can I understand myself, improve myself, and ultimately become my ideal self? In this context, “ideal humanity” and “ideal self” are interconnected, although the Chinese artistic tradition places greater emphasis on “ideal humanity.” Sources of the concept of “ideal humanity” in both Chinese and Western philosophy include Kant’s (2000, p. 24) notion of “the feeling of the beauty and the dignity of human nature,” as well as Wang Guowei’s idea of the “complete person,” which considers the comprehensive development and harmony of body and spirit, alongside his emphasis on the unity of truth, goodness, and beauty (Wang, 2014b, pp. 45–47). It is generally believed that, Chinese humanity can be understood to exist on four levels: the natural person, ordinary person, noble person, and sage (Wang et al., 2019). Since the function of the “sublime” is not a fixed or restrictive concept but rather a dynamic force that inspires free thought in the mind (Shaw, 2017, p. 150), the modern interpretation of ideal humanity extends beyond a select few who are gifted or sages. It encompasses a process of opening oneself to believe in, discover, and awaken individual potential. The pursuit of the ideal becomes a means of freely realizing the sublime goal of elevating one’s mind. As long as they maintain the sincerity of character and extensive knowledge, ordinary people can achieve the essence of sublime beauty (Wang, 2014a). This fluid and multifaceted approach to cognition includes different experiences of reason and intuition and is interconnected with the rich experiences of individual ideal humanity: one’s innate nature, emotional expression, self-discipline, mental state, and specific moods (Shusterman, 2013).

**Figure 1:**
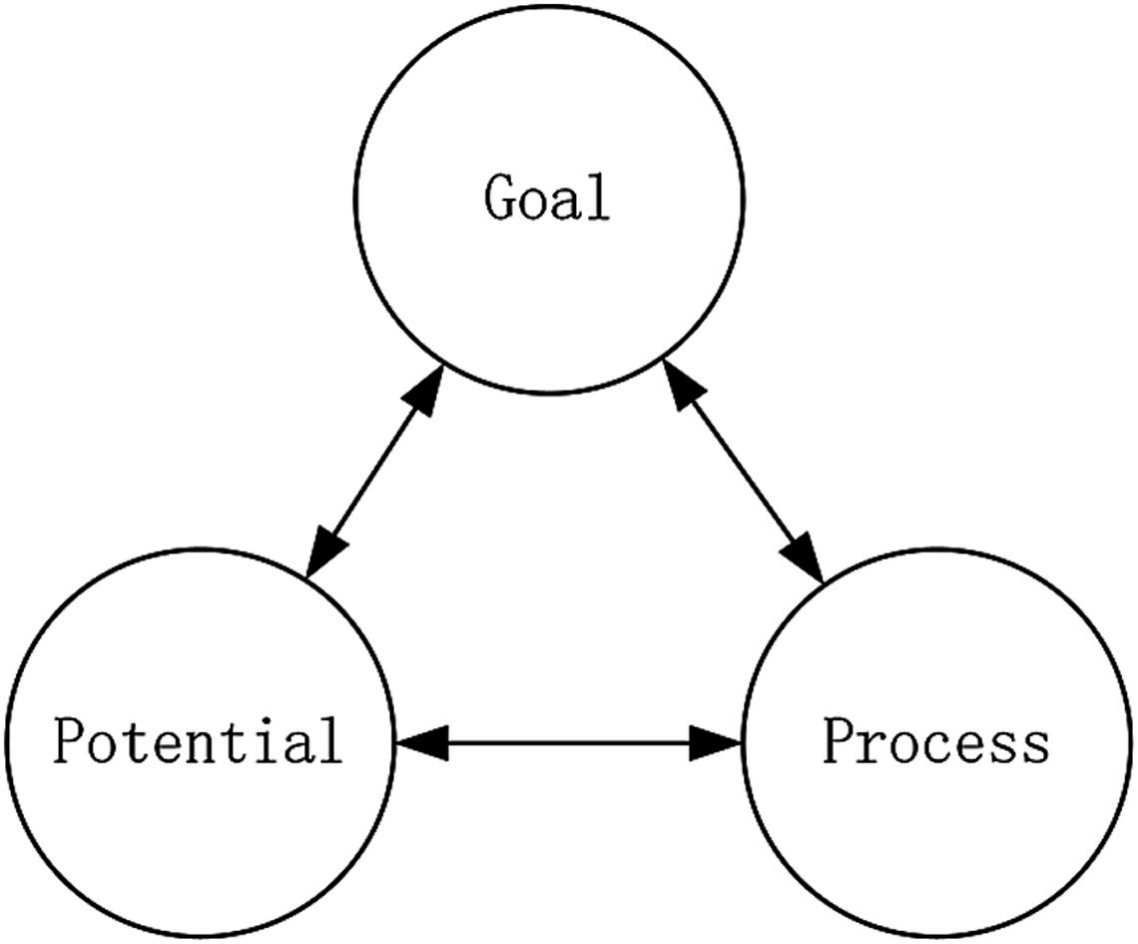
The research model.

**Figure 2:**
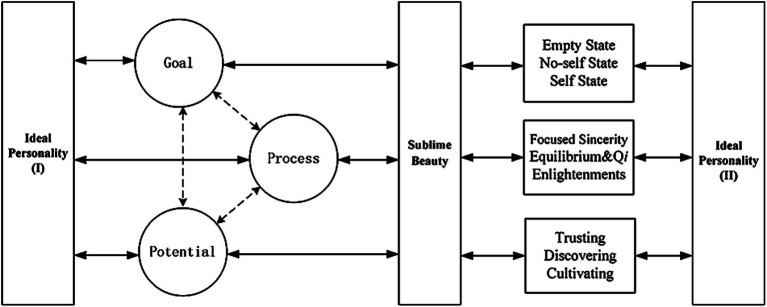
The relationship between ideal humanity and sublime beauty.

This paper employs the model of “potential-process-goal” (see Figure 1) to analyze, from cognition to practical implementation, the interactive relationship between the mind and sublime beauty, with a focus on the concept of ideal humanity. This chapter summarizes specific features of thought and the modern values of the Chinese artistic mind and sublime beauty. Specifically, potential refers to an individual’s unique talents and disposition, encompassing universal nervous system-based and cognitive approaches that form the foundation of the mind to facilitate the creation of the sublime beauty of art and the realization of ideal humanity. Process refers to specific cognition and practical actions within the mind of a subject that lead to the pursuit of sublime beauty. Within the specific practical process, the diversity of individual backgrounds and contexts inevitably sparks each individual’s unique innate potential and diverse ideal humanistic goals. Value goals are the highest form of the mind in humanity’s quest for sublime meaning. These goals are not static but evolve through continuous interaction with one’s potential and processes, integrating historical cognitive experiences with personal artistic creation. In terms of the relationship between the three concepts, “potential-process-goal” follows a cognitive successive sequence. However, because of the complex and ever-changing nature of the relationship between the mind and sublime beauty, these three elements are also constantly evolving and combined to construct the ideal humanity of different individuals, and even the diverse ideal humanities that may develop over the course of an individual’s life (see Figure 2).

The application value of this article’s “potential-process-goal” lies in three aspects: (i) Systematicity. This framework comprehensively summarizes the three stages of the relationship between humans and sublime beauty—human potential, the practice process, and the ideal goal—providing empirical references for future research. (ii) Dynamicity. It avoids rigid frameworks by positioning “ideal humanity” as the main body, thereby explaining the dynamic interplay among these three stages and analyzing the organic functioning of the human mind. (iii) Openness. It transcends disciplinary and cultural boundaries, integrating interdisciplinary and cross-cultural perspectives while accommodating various possibilities.

To illustrate the application value of this research model in exploring the relationship between the mind and sublime beauty, we examine the case of the Chinese painter Qi Baishi (齊白石,1864–1957) and his sustained artistic creativity. Despite coming from a poor background and lacking formal art education, Qi relied on self-study, expanded his knowledge base, sought mentorship from renowned artists, and continuously explored his potential. Notably, he pursued new artistic styles well into his fifties and ultimately achieved success across multiple fields, including poetry, calligraphy, painting, and engraving (Olivová, 2011).

**Figure 3:**
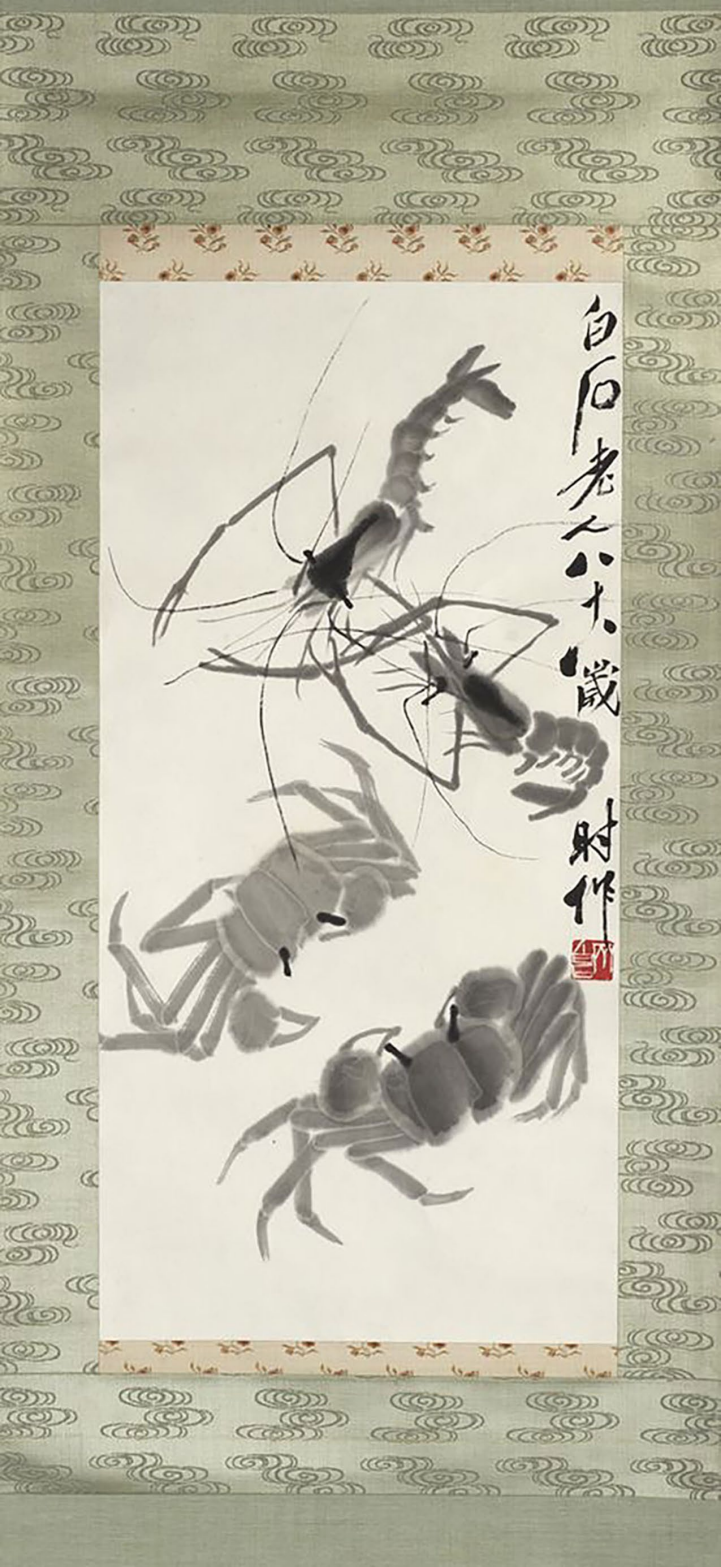
Shrimps and Crabs. Qi Baishi. Princeton University Art Museum (https://artmuseum.princeton.edu/collections/objects/31136).

The “potential-process-goal” research model presented in this article offers a systematic perspective for integrating diverse viewpoints and analyzing the intricate interactions between ideal humanity and sublime beauty in Qi Baishi’s pursuit of literati painting (文人畫; Mastandrea et al., 2019). This approach aims to restore the artist to his authentic “ideal humanity.” For instance, the two crabs and two shrimps (see Figure 3), which is a common theme that emerged after Qi’s rise to fame, showcasing his ability to extract beauty from the natural world. This sublime creative state must be understood through the complex interplay of his natural endowments (potential), his long-term dedication to exploration (process), and the universal values embodied in literati painting (goal). Any omission of these components may hinder a comprehensive understanding of his “ideal personality.” Therefore, this article will explore the specific relationship between ideal humanity and sublime beauty through the three dimensions of potential, process and goal, combining theoretical insights with visual analysis of paintings.

## 2 Relationships with the dimension of potential: trusting, discovering, and cultivating the self

### 2.1 Fully trusting one’s own potential

In terms of ontological cognition, Chinese artistic thought highly values the innate potential of the individual, believing that every person contains the seed of “ideal humanity” and that this potential can be realized through the pursuit of the ideal. This provides both cognitive foundation and psychological implications for the creative process and values the artistic practice (see Figure 2). This ideal potential is primarily reflected in the equal standing of human beings within metaphysical existence and includes an affirmation of the beauty of life. The *I Ching* places humans alongside heaven and earth to form the “Three Powers (三才; Kong, 1980, p. 90),” and Laozi (Gao, 1996, p. 351) emphasizes the equal standing of human beings alongside the *Dao*, heaven, and earth, calling them the “Four Greats (四大).” Mencius (Zhu, 2016, p. 345) asserts that “anyone can be like the legendary sages Yao and Shun” (人皆可以为堯舜). These views all stress the unity between human beings and the sublime beauty of existence within the flux of life, promoting the belief that human beings have great potential to be equal to the sacred and sublime beauty, which is the spiritual origin of Chinese artistic thought. In comparison, Kant (2000, pp. 147-148) also posited that the sublime is “only in our mind,” advocating for the use of reason to judge the powerful forces of nature without fear. He emphasized the significance of human subjectivity in the process of cognition. Consequently, both Chinese and Western thought recognize the value of “potential” within this research framework.

The traditional Chinese understanding of self-potential emphasizes the integration of the universal nature of ideal humanity with the uniqueness of individual potential. In other words, although each person’s innate talents and disposition are unique, anyone can attain universal ideal humanity by realizing their potential (Xu, 2005, p. 74). *The Doctrine of the Mean* (中庸) stresses that an individual’s special potential is the result of an individual’s innate gifts and that one should trust and follow their own nature, which is to follow the sublime *Dao*. This process and approach are central to spiritual education (Zhu, 2016, pp. 50–51). This cognitive characteristic stems from the Chinese belief that one’s heart, or nature, is the core of one’s being, capable of encompassing the entire universe and the sublime beauty within it, without needing to seek out metaphysical concepts beyond the spirit. In other words, Chinese philosophy firmly believes in the unique resonance between individual spiritual potential and the natural universe: through pure perception and self-cultivation, one can reveal the infinite sublime possibilities contained within the spirit. Correspondingly, when analyzing the five most productive sources of sublime, Longinus (1995, p. 181) emphasized that the power of grand conceptions and inspiration of vehement emotion “are for the most part congenital.” Similarly, Burke (1990, p. 48) also found that the “wisdom of Creator” exists in every part of our body. From this, it can be seen that the affirmation of sublime potential in China and the West is interlinked, but they have different understandings of the ontological source of “potential.” This difference and diversity of sublime potential in China and the West is also the theoretical basis of this article’s research model.

**Figure 4:**
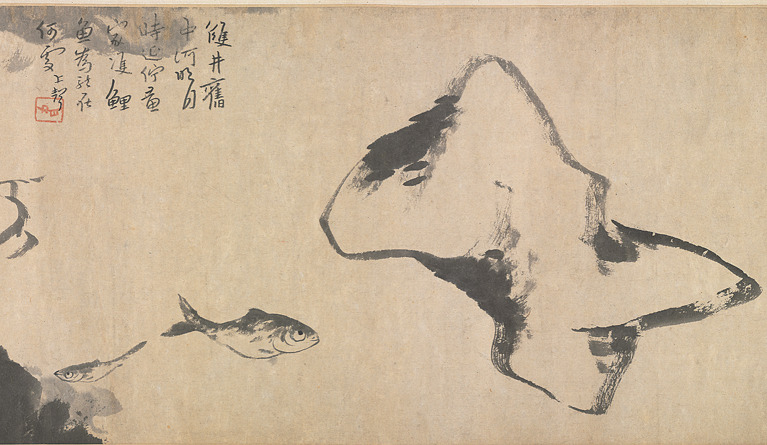
Fish and Rocks (detail). Bada Shanren. The Cleveland Museum of Art (https://www.clevelandart.org/art/1953.247).

In terms of modern value, this positive affirmation of the human ability to discover one’s sublime potential provides ordinary individuals with confidence in life, helping those undergoing psychological healing or facing physical disabilities to realize the infinite possibilities of self-recovery: the spiritual potential within the self is internal and limitless, unconstrained by external physical or material limitations. Psychological research shows that the “self-rewarding nature of aesthetic experience” in art appreciation can enhance an individual’s happiness and creativity (Mastandrea et al., 2019). The applicability of the ideal humanity mentioned by Zhuangzi incorporates not only immortal beings but also those with physical disabilities (Guo, 1961, p. 180). Only by fully trusting in oneself and relinquishing excessive worries can an individual manage one’s own organic being (Maslow, 1971, p. 13), appreciate the silent beauty of heaven and earth, and ultimately enter the sublime realm of the mind. Similarly, experiments revealed that participants’ emotional expectations, predictions, and anticipations of peak aesthetic pleasure also led to dopamine release (Salimpoor et al., 2011). Therefore, people’s belief in their potential to experience sublime beauty has both psychological and physiological positive effects. The painting *Fish and Rocks* (魚石圖; see Figure 4) by Bada Shanren (八大山人), for example, depicts the image of a fish rolling its eyes to demonstrate the artist’s ability to empathize with all things through his artistic perception. However, as he seems unable to forget his identity as a remnant of the fallen Ming dynasty, the painting’s large areas of blank space, combined with nervous, erratic brushstrokes, create a contrast that exposes his contradictory emotions—his desire to forget the self while simultaneously seeking to express it through others. This reveals the artist’s loneliness, conflicting emotions, and cognitive barriers. The pure beauty of the black and white contrast in the painting also forms an interesting echo with Burke’s (1990, pp. 73–74) view that both “light” and “darkness” can bring a sense of sublimity. However, the difference is that one of the sources of this sublime aesthetic taste in Chinese painting is the pursuit of the concept of interaction between *yin* (陰) and *yang* (陽), a set of concepts that emphasize the unity of opposites. Burke’s the extreme harmony of light and darkness is related to “the divine presence.”

### 2.2 Discovering one’s potential through art

Based on this understanding of potential, traditional Chinese thought advocates for the integration of life and art as a means to discover and realize individual potential, progressively approaching universal ideal humanity through various personal processes. The method for discovering the self is to unify life, art, and the sublime “*Dao* (Way)”: The “Way” is not separated from everyday life; through the consistent practice of skills, one can discover the unique connection between individual potential and the sublime, thereby gradually constructing one’s own ideal humanity. With the help of exotic cultures, including Eastern thought, Maslow (1970, pp. 83-90) discovered that “the sacred is in the ordinary, that it is to be found in one’s daily life.” In early Chinese art forms, the ideal goal of discovering individual potential was the sages’ ability to participate in the creation of heaven and earth for the benefit of all things. From a modern perspective, this human ability may seem more mysterious. The core lies in humans striving to grasp the sublime laws of the universe through specific “artistic forms,” thereby maximizing personal advantages, avoiding harm, perfecting oneself, governing society and all earthly things, and promoting the “*sheng-sheng*” process. Psychological experiments have shown that this sublime experience is not only very common, but can also lead to a large number of positive experiences, including“high reported pleasure (i.e., feeling of amusement, sensuality, mindfulness, sublime, and sense of beauty), tension (surprise and powerful force), bodily arousal, and self-awareness as well as transformation or insight (i.e., denoted by feeling of profundity and realization)”(Pelowski et al., 2021).

For example, the Eight Trigrams (八卦, *Ba gua*) of the *I Ching* is both a scientific representation of early Chinese cosmology and an artistic symbol. Specifically, the Eight Trigrams describe the eight basic elements of the universe, with examples including heaven, earth, thunder, and wind. These elements are interrelated, producing 64 patterns of cosmic change and human wisdom extending to 384 possible life circumstances and behavioral experiences. While the symbolic art of the *I Ching* may seem complex and difficult for ordinary people to grasp, through awe, diligent study and sincerity, anyone can unlock the cognitive “potential” to understand these symbols. Moreover, each individual’s unique gifts and fate can lead to a better understanding of the laws of these patterns through personal effort. Comparatively speaking, although Longinus (1995, p. 185) also emphasized that lofty thoughts (previously expressed as “the power of grand conceptions”) come from talent, he also advocated that lofty thoughts need to be acquired through continuous training of mind. Once one masters this ability, they can perceive unknown possibilities and participate in the mechanisms of the sublime *Dao* of the universe for the benefit of all life. At this point, one approaches the state of ideal humanity: maintaining self-reflection and daily vigilance, moderating one’s desires, and striving toward the sage-like goal of contributing to the positive development of society.

### 2.3 Three relationships for cultivating one’s potential

**Figure 5:**
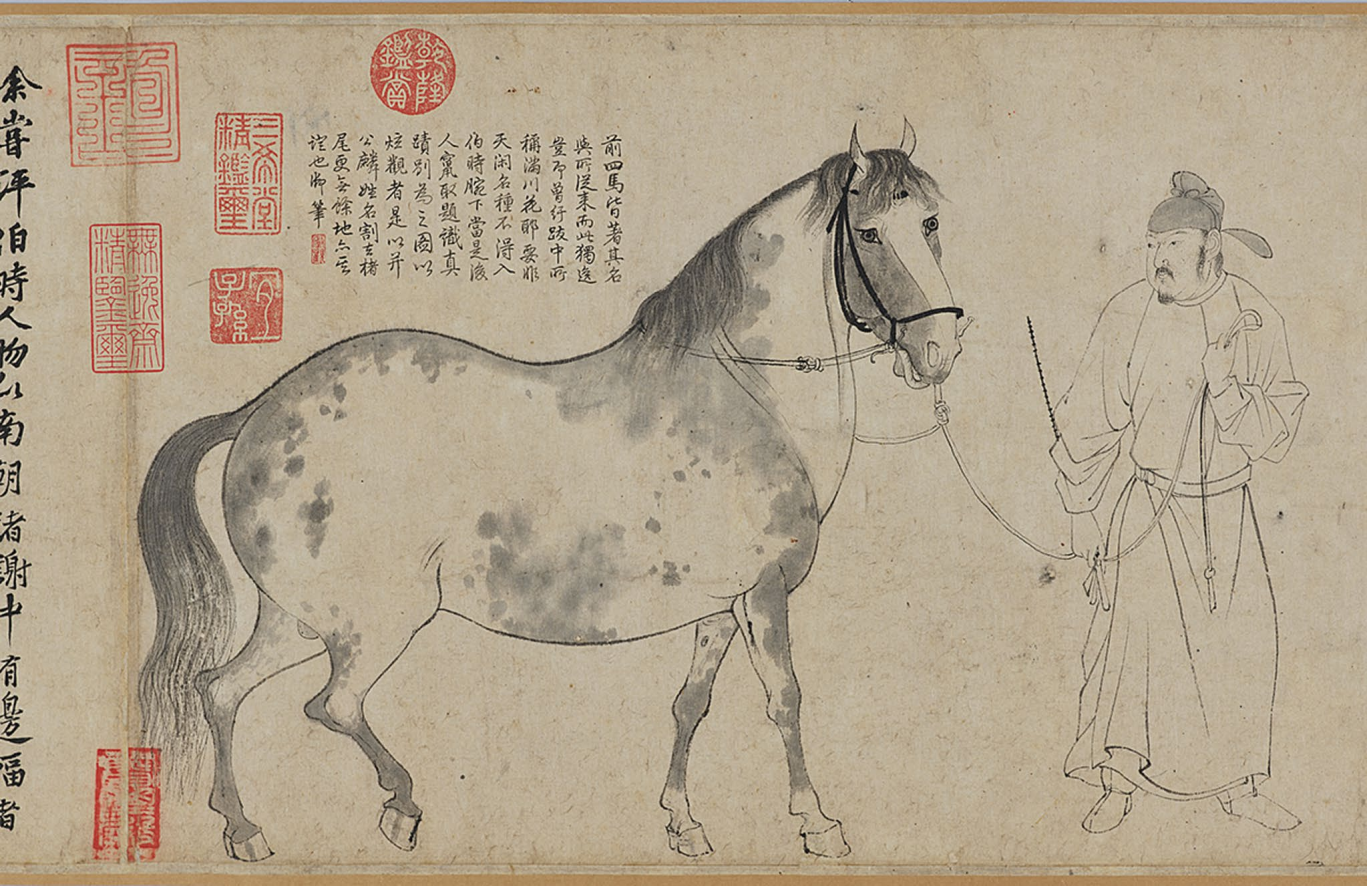
Five Horses Scroll (detail). Li Gonglin. Integrated Collections Database of the National Institutes for Cultural Heritage, Japan (https://colbase.nich.go.jp/collection_items/tnm/TA-694?locale=ja).

It is from this spiritual potential that Chinese art continually absorbs subtle and varied spiritual experiences, gradually cultivating an understanding of the differing relationships between one’s spirit and the Creator of heaven and earth. First, Zhangzao (張璪)’s view of “Draw artistic inspiration from both imitating natural transformation and grasping creativity of one’s mind” (外師造化, 中得心源; Zhang, 2001, p. 91) reflects the process of learning from nature to activate the spiritual potential of the individual and realize artistic creation. This cognitive approach has two dimensions: From an ontological perspective, elevating one’s mind to a state of participation in and promoting the order of heaven and earth for the benefit of humanity maximizes individual potential, enabling the spirit to establish a harmonious relationship with sublime beauty through intuition and imagination (Zhou, 2005, p. 88). From a practical cognitive standpoint, to learn from nature, one must observe the smallest details—such as a flower’s petals or a leaf’s veins—and observe both familiar and unfamiliar (Zhou, 2005, p. 102). Through the “reason (理, *li*)” of different objects or physical phenomena, it is possible to understand the life force and operational laws behind the growth of all things. Through rational and sensory experiences, one can transform this knowledge into artistic creation (Sullivan, 2015, p. 67). Chinese painting, for instance, emphasizes “expressing spirit through form.” In Li Gonglin (李公麟)’s *Five Horses Scroll* (五馬圖卷; see Figure 5), simple line drawings and subtle coloring capture the natural form of horses. The horses’ eyes express a human-like spirit and temperament, evoking an otherworldly beauty. The strong, round, and flowing lines of the horses’ backs demonstrate the artist’s mastery of the natural order.

**Figure 6:**
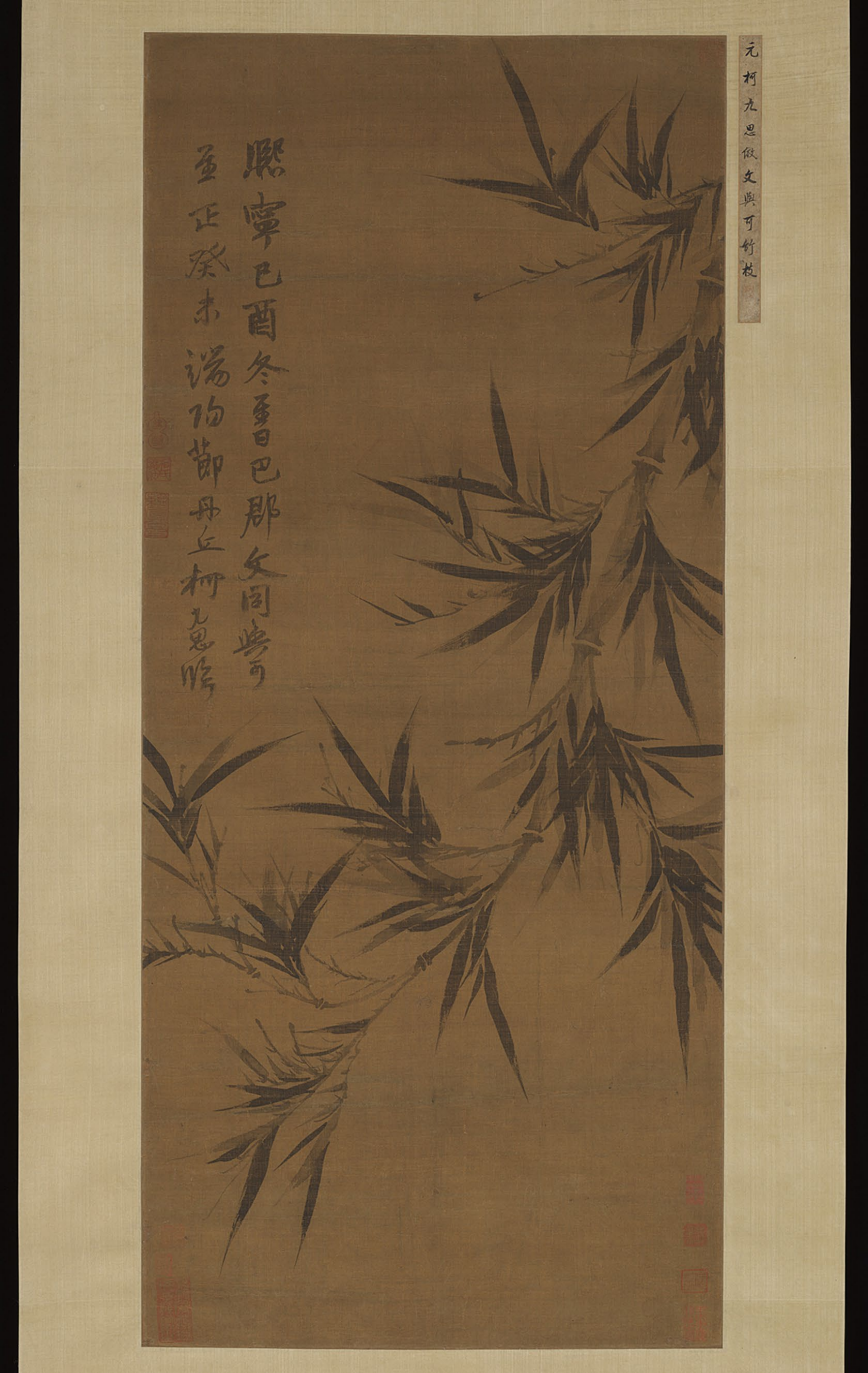
Bamboo Copied after Wen Tong. Ke Jiusi. The Metropolitan Museum of Art (https://www.metmuseum.org/art/collection/search/39546).

Second, the concept of “seizing nature (夺造化)” emphasizes the stimulation of human cognitive potential beyond the superficial to reveal the inner “artistic charm” of sublime beauty and transform it into artistic creation. “Seizing nature” highlights the assertion of self-subjectivity and can transcend mere visible surface beauty and capture the inner beauty created by sublime nature (Wang, 1982, p. 2). When one moves from learning from nature to seizing nature, artistic creation is no longer about imitating the outward forms of nature but takes the potential of the artist as its essence, respecting the innate talents and disposition of the artist and harnessing natural potential to realize the cognition and perception of sublime beauty. Finally, the artist constructs their own unique form of sublime beauty. For example, Ke Jiusi (柯九思) praised Wen Tong (文同) for “seizing nature” in his depictions of bamboo (Zong, 1985, p. 136). In his *Bamboo Copied after Wen Tong* (臨文同墨竹圖; see Figure 6), Ke not only emulated Wen’s style but also transcended pure replication, focusing on using simple ink brush strokes to capture the graceful yet resilient spirit of bamboo. According to Seligman and Csikszentmihalyi (2000), this kind of stimulation includes “positive individual traits” of “high talent” that “move individuals toward better citizenship,” ultimately aiming to achieve “ideal humanity.”

**Figure 7:**
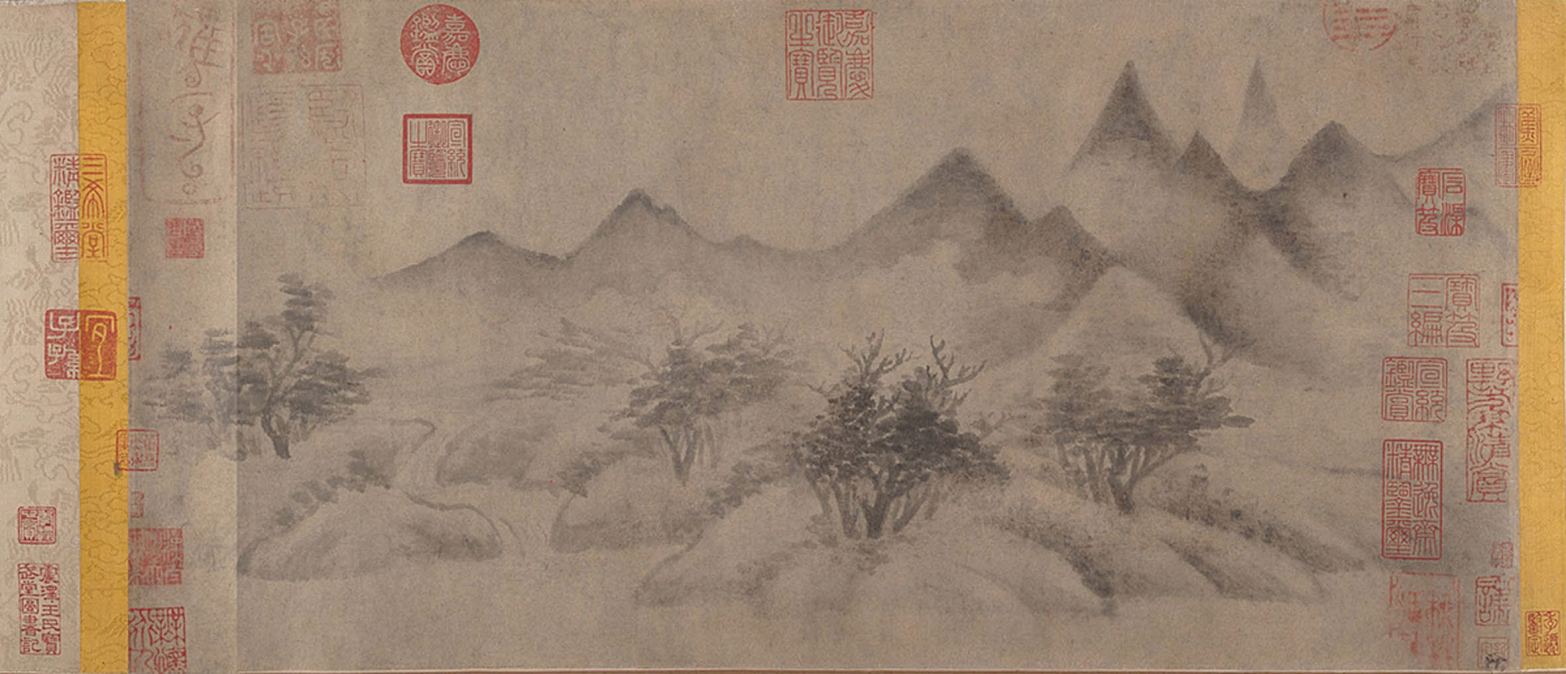
Cloudy Mountains. Mi Youren. Metropolitan Museum of Art (https://www.metmuseum.org/art/collection/search/40007).

Finally, “beguiling nature (欺造化)” asserts that the artist’s mind is superior to the nature of heaven and earth, showcasing the wonderful creativity and sublime beauty of the artist’s true inner psyche. This practice, undertaken by literati outside of their political work and literary cultivation, is referred to as “ink play (墨戲),” a form of creative expression born from the mind. The artist does not need to be familiar with traditional painting techniques; instead, they simply follow their own spiritual potential, including their temperament, interests, and moods, showing the true state of one’s mind in a casual way, one can bring out the beauty of one’s suppressed humanity by daily desires. Schellekens (2007, p. 109) believes that the sublime can lead us to contact “what amounts to the source and possibility of our morality.” Cohen et al. (2008) pointed out that this unconscious experience of profound beauty “emphasis on circumstances more so than on the self being in control and an emphasis on understanding.” In short, as long as everyone returns to their own “ideal humanity,” the creation process of sublime beauty has infinite possibilities. For example, Mi Youren (米友仁)’s *Cloudy Mountains* (雲山圖; see Figure 7) uses wet brush techniques to create a misty, ethereal effect that evokes the mysterious depths and ever-changing qualities of the artist’s mind. Empirical research points out that compared with Western realistic oil paintings, viewers of traditional Chinese shanshui (山水; landscape) paintings “experienced greater levels of relaxation and mind wandering” (Wang et al., 2015). This may suggest the difference between the sublime aesthetic psychology of China and the West: Chinese tradition focuses on irrational feelings, while Western oil paintings tend to be rational cognition.

In summary, within the “potential-process-goal” research model, the dimension of potential emphasizes that the creative subject must trust oneself, discover oneself and cultivate oneself in the “process” of freedom and openness, and finally reach everyone’s “goal” by realizing the ideal humanity in “potential” (see Table 1).

**Table 1:** Summary of key arguments of relationships with the dimension of potential.

Potential stages	The thoughts of recognize sublime beauty	The ways to achieve sublime beauty
Trusting one’s own potential	Every individual potential contains the seeds of “ideal humanity”	Maintain awe, sincerity and diligence to realize one’s ideal humanity
Discovering one’s potential	The process of realizing the “ideal humanity” in one’s own potential is different for everyone	Through the consistent practice of skills, one can discover the unique connection between individual potential and the sublime
Cultivating one’s potential	Imitating nature: learn from nature, which contains sublime entities and embodiments	Observe and grasp the inner truth of objects, and display the sublime beauty of nature through art
	Seizing nature: stimulate cognitive potential, transcend appearances, and grasp the inner beauty created by sublime nature	From the painter’s personal potential (talent and temperament), he constructs his own unique sublime beauty
	Beguiling nature: the sublime beauty of ideal humanity’s true soul surpasses that of nature	Showing the true state of one’s mind, one can bring out the beauty of one’s suppressed humanity

## 3 Relationships with the dimension of process: the ideal mental state, ways of thinking, and two cognitive approaches

### 3.1 Mentality of focused sincerity in artistic creation

In terms of the ideal mental state during the creative process, Chinese art highlights the artist’s focused sincerity (精誠專一; see Figure 2). This state is one in which the individual self merges seamlessly with the sublime. In this state, the artist’s mind remains undistracted by other visible things and is instead fully concentrated and immersed in the evolving form of the artwork. This “intense and focused concentration on what one is doing in the present moment” (Csikszentmihalyi, 2014, p. 240) is also one of the “flow” characteristics of positive psychology. From a cognitive perspective, when perceiving the sublime beauty of nature, the artist’s emotions and imagination are gradually activated. The artist must then transcend the outward appearance of things and project their mind onto the essence of the object, unlocking their boundless imaginative potential with an undistracted mind.

**Figure 8:**
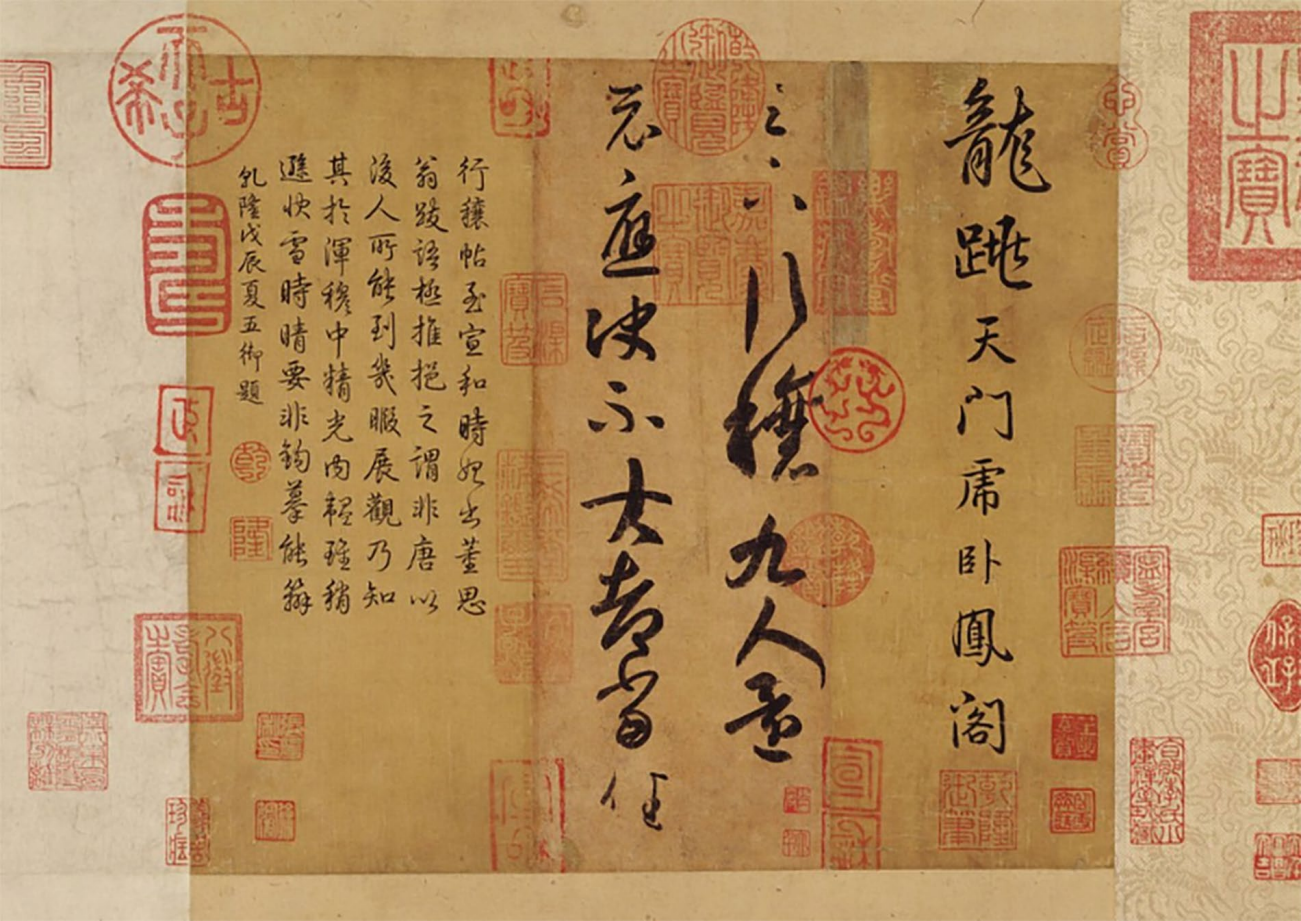
Ritual to Pray for Good Harvest. Wang Xizhi. Princeton University Art Museum (https://artmuseum.princeton.edu/collections/objects/35203).

Focused sincerity refers to the complete unity of the self with heaven and earth, through which inspiration for sublime beauty can be drawn. This mental state emphasizes the stable relationship between the artist’s mind and the sublime being, requiring the creator to maintain purity and sincerity throughout the artistic process to ensure the continuation of inspiration. This kind of sincerity that integrates the sublime and ideal humanity is a common psychological state in both China and the West (Kant, 2000, p. 24). A prolonged period of mental focus within the mind of a subject enables the artist’s sensibility, thoughtfulness, and creativity to be greatly enhanced, enabling them to form extraordinary connections with all things in the world. Burke (1990, pp. 124-125) also believes that only consistent labor can lead to intense pain and inspire a sense of the sublime. Hegel (1975, p. 375) attributes the psychological satisfaction of obtaining the sublime to “in this recognition of the nullity of things and in the exaltation and praise of God.” In contrast, Chinese artists’description of the source of the sublime has its own traditional characteristics: Zheng Xie (鄭燮) believes that, in such a state, one can even receive creative inspiration from divine beings, ghosts, humans, and objects (Zhou, 2005, p. 149). In Chinese calligraphy, there is a reverence for the practice of “one-stroke calligraphy (一筆書)” or “one-stroke painting (一筆畫),” which emphasizes that the brush should never leave the paper. This consistent engagement with the medium allows the artist to pursue a constant and authentic mental state, along with the mysterious creativity inherent within improvisation. Because Chinese calligraphy and painting typically use tools like brush and ink, and because the ink must be re-dipped after several strokes, a single stroke can rarely be used to complete too many characters. For example, in “Sage of Calligraphy” Wang Xizhi (王羲之)’s famous calligraphy *Ritual to Pray for Good Harvest* (行穰帖; see Figure 8), the entire text comprises 15 characters in two columns. The third character in the right column, “行,” is seamlessly connected with the fourth character, “穰.” At the same time, examining the space between the characters—such as the fifth and sixth characters of the right column, “九” and “人”—there is a distinct up-and-down echo of the brush between the strokes, showing the temporal flow of the writing process. Additionally, the final four characters of the second column (“大者當任”) retain the echoed relationship of the previous strokes while spreading out from side-to-side, opening up the freedom of space and allowing for the dynamic expression of one’s individual life and thought. As a fragment of a daily letter, this piece exemplifies the writer’s profound mental focus while remaining natural and relaxed, blending the mind, brushwork, and the sublime *Dao* into a unified whole. In this psychological process of focusing on the sublime, “bodily feelings of power, solidity or aloofness can be called up quite automatically” (Cochrane, 2012).

### 3.2 Creative thinking as a balance between “equilibrium” and “*qi*”

In the realm of creative thinking, Chinese artistic thought advocates for the “equilibrium (度, *du*)” that harmonizes creative thinking with the rational expression of emotions and the self-cultivation of “*qi* (氣)” in the artist, to maintain the balance of the Doctrine of the Mean and the “method of no-method (無法之法),” bringing one closer to sublime beauty (see Figure 2). “Equilibrium” in Chinese art refers to the inner morality and social etiquette that govern the expression of emotions in line with ideal humanity. This equilibrium is influenced by both inherent potential and the pursuit of sublime goals. Chinese art emphasizes the expression of emotions and maintains a tension between internal impulses and external constraints. This concept is rooted in Confucian thought, specifically the Doctrine of the Mean: first, one becomes aware of the internal “unexpressed” emotional state, and then, once emotions are put into artistic form, they must align with the value forms of ideal humanity (Zhu, 2016, p. 18). This self-reflective “method of equilibrium (法度, *fa du*)” mirrors Chinese spiritual practice in the pursuit of sublime beauty (Zhang, 2012, pp. 47–51).

In addition, “*qi*” in Chinese art refers to the pursued self-cultivation of ideal humanity and relatively free artistic expression. “*Qi*” represents the unity between the inner mind of the artist and their outward expression. The “*qi*” of ideal humanity allows one to harmonize with the sublime beauty of heaven and earth, balance rational cognition and intuitive perception, and adapt to the ever-changing affairs of life. When the artist “withdraws one’s *qi* and returns to the mind,” meaning that they reflect inwardly rather than focus on the other, their creative expression naturally flows from their regular self-cultivation, without the need for deliberate contrivance. In Chinese calligraphy and painting, the “stroke (綫)” is the focal point, with each stroke embodying the complex qualities of the artist’s ideal humanity. The thickness, dryness, speed, and other variations in the brushstrokes record changes in the artist’s “*qi*”—the artistic manifestation of their pursuit of sublime beauty. Since “*qi*” is flexible, open, and individual, it is simultaneously free, conscious, and understands the discipline of “method of equilibrium” in artistic practice. At this stage, the artist’s mind is in harmony with the *qi*. They know when to stop and when to move, guided by a harmonious balance of rationality and emotion. Moreover, because this “*qi*” is in tune with the changes of heaven and earth, the artist, in their unintentional pursuit of uniqueness, may create extraordinary expressions and sublime beauty that transcend human effort (Zhou, 2005, p. 148).

### 3.3 Gradual and sudden enlightenment in cognitive approaches

In terms of cognitive approaches, ideal humanity in Chinese art emphasizes the gradual accumulation of embodied cognitive experience (漸悟, *jian wu*, gradual enlightenment) and the sudden moments of insight that reveal personal talents (頓悟, *dun wu*, sudden enlightenment), both of which lead to the realization of sublime beauty (see Figure 2). Dong Qichang (董其昌) divided Chinese painting into two schools—the southern and the northern—based on these two cognitive approaches, criticizing the gradual enlightenment of meticulous brushwork while praising sudden enlightenment. “*Wu* (悟; enlightenment),” an important concept in Chinese cognition, transcends rational cognitive states. The true enlightenment is “the full awakening of the total personality to reality,” which emphasizes that one should no longer regard the object of cognition as an object, but respond to the world as a whole person (Fromm et al., 1960, pp. 115–116). Experimental neuroaesthetics reveal that when humans appreciate the sublime and beauty, they experience a complex state of mind that combines “the experiential, sensory and cognitive element” (Ishizu and Zeki, 2014). This psychological state is associated with “enlightenment (*wu*).”

First, “gradual enlightenment” in artistic education is described as “wandering (遊, *you*),” and emphasizes the importance of respecting the basic laws of embodied cognition. Cognition often arises from the accumulation of surface-level knowledge over time, eventually leading to the grasping of true esthetic perception. The ideal outcome of this self-cultivation is that the artist, in the process of “unknowing,” enters the realm of the sage (Zhu, 2016, p. 94). This type of gradual enlightenment education on artistic self-discovery has been applied internationally. Research has revealed that many non-Asian American students gradually discover the sublime beauty in their inner nature through the practice of one-brush and one-stroke calligraphy, learn to observe and express themselves (Tu, 2010), and even feel connected to the sublime beauty of the world.

**Figure 9:**
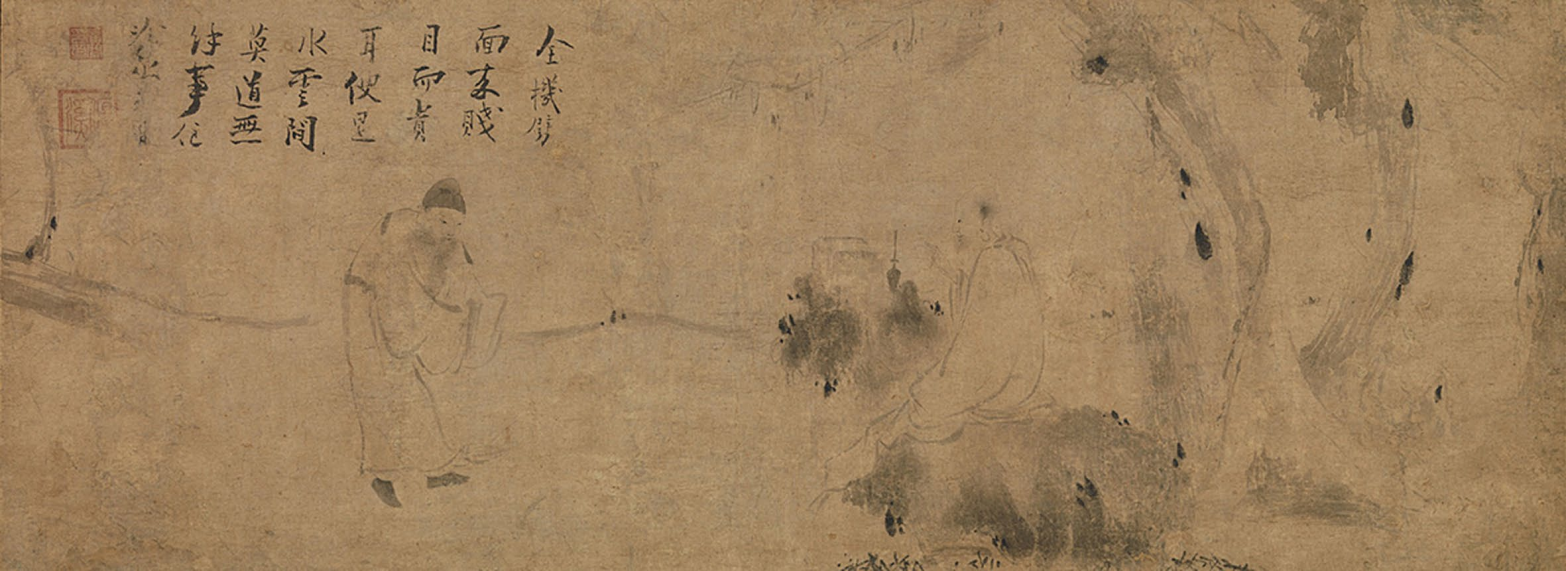
Meeting between Yaoshan and Li Ao. Attributed to Zhiweng Chinese. The Metropolitan Museum of Art (https://www.metmuseum.org/art/collection/search/40278).

In contrast, “sudden enlightenment” focuses on individual creative talents and the serendipitous, transformative moments of insight during the cognitive process. Chinese literati painting, especially Zen painting, emphasizes the concise summarization that goes beyond the appearance in brushwork, and intuitively convey the experience of sudden enlightenment, thus transcending gradual enlightenment to attain the sublime. Gradual enlightenment is not without its flaws; the long-term embodied experience, without metaphysical reflection or intervention, may cause the individual to become lost in the details of representation. At this point, a teacher may need to give a “blow to the head (當頭棒喝; Loy, 1992)” to open the mind of the learner, leading to an enlightenment in which the individual transcends time, space, and causality, even transcending the boundaries of the self and the object and the self and the other, merging with the universe and attaining the sublime (Li, 2017, pp. 191–193). Correspondingly, this theory of the epiphany of beauty is also supported by the results of modern neuroaesthetic experiments: the aesthetic experience of painting and music is organically linked to mOFC (medial orbito-frontal cortex). Therefore, the researchers revised Burke’s definition of beauty as “mechanical,” highlighting the mental advantage of people in understanding beauty (Ishizu and Zeki, 2014). The creators of Chinese paintings, drawing on their own experiences of sudden enlightenment, transform everything they see into artistic images through their brushes. At this moment, any visible image painted is merely a temporary “reflection” of the world received by the artist (or the enlightened Zen practitioner) through a clear and enlightened mind (Xu, 2012, p. 113). For instance, in the famous painting *Meeting between Yaoshan and Li Ao* (藥山李翱問道圖; see Figure 9), attributed to Zhiweng (直翁), Confucian scholar Li Ao asks the Zen master Yao Shan about the sublime “*Dao*.” The master’s response with a finger pointed to the sky, “(Be like) water in a bottle, a cloud in the blue sky (雲在青天水在瓶),” seems ambiguous at first, but it is in fact a moment of sudden enlightenment: whether clouds or water, all things exist in their proper place, just as they are. To attain the sublime “*Dao*,” one must transcend complex cognitive concepts, such as cognition, mind, logical thinking, intellect, self, etc. the false dichotomy of right and wrong, and return to one’s true heart, like clouds and water, effortlessly flowing in their natural state. From a cognitive perspective, “not only intellect, but any authoritative concept or figure, restricts the spontaneity of experience” (Fromm et al., 1960, p. 120). Because in the view of Buddhism, these cognitive concepts are only steps to enter the sublime realm of colorless and invisible, but they still have limitations. Only by transcending cognition and its concepts can we achieve true transcendence and enter the realm of freedom.

In short, under the research model of “potential-process-goal,” the above discussion shows the interactive relationship between the mind and sublime beauty of Chinese artists in the process dimension: the ideal mental state, the creative thinking and cognitive approaches. From a comparative perspective, the contemporary value of Chinese artistic thought needs to be further explained in order to better tap the potential of people of different cultures and inspire each individual to constantly adjust cognitive goals, thereby reflecting on the process of self-existence (see Table 2).

**Table 2:** Summary of key arguments of relationships with the dimension of process.

Creative process	The thoughts of recognize sublime beauty	The ways to achieve sublime beauty
The ideal mental state	Focused sincerity: the individual self merges seamlessly with the sublime beauty	The artist must then transcend the outward appearance of things and project their mind onto the essence of the object, unlocking their boundless imaginative potential with an undistracted mind
The creative thinking	Equilibrium: self-emotional expression is in line with the inner morality and social etiquette of ideal human nature	Chinese calligraphy and painting emphasis on the use “center point” of the brush in a controlled and correct manner, inspiring and elevating the mind into a sublime state
	*Qi*: the unity between the inner mind of the artist and their outward expression	The artist’s mind is in harmony with the *qi*, and realizing the sublime beauty of integrating “equilibrium”
Cognitive approaches	Gradual enlightenment: emphasizes the gradual accumulation of embodied cognitive experience to the grasping of true esthetic perception	Gradually discover the sublime beauty in their inner nature through the practice of one-brush and one-stroke calligraphy, learn to observe and express themselves
	Sudden enlightenment: focuses on individual creative talents and the serendipitous, transformative moments of insight during the cognitive process	Focus on concise summarization that goes beyond the appearance through brush and ink, and intuitively convey the experience of instant enlightenment

## 4 Dimension of goals: the “self state,” “no-self state,” and “empty state”

The sublime goal of ideal humanity in Chinese art is encapsulated in the core concept of “境界 (state, *jing jie*; Zong, 2005, p. 118),” which refers to the goal of creating sublime beauty through the realization of one’s ideal humanity. The individual potential, creative process, and value goals of each artist influence their own ideal “state.” Based on the main schools of Chinese artistic thought, the pursuit of ideal humanity in Chinese art encompasses three states: the “self state,” the “no-self state,” and the “empty state.” (see Figure 2) In the “potential-process-goal” research model presented in this article, “goals” serve to motivate artists and their creative endeavors, thereby enhancing their ability to stimulate their own “potential,” maintain focus on the creative “process,” and continually approach sublime beauty. According to research findings in positive psychology, this reflects the actual function of “highly valued goals” (Carr, 2022, p. 31). Additionally, Cupchik et al. (2009) argue that the control of human aesthetic cognitive goals and the actual perception process are mutually interactive, thereby jointly enhancing the aesthetic experience. Consequently, it is essential to uphold the systematic interaction within the research model outlined in this article.

### 4.1 The “self state”

The “self state” (有我之境, *you wo zhi jing*) refers to the conscious pursuit and expression of one’s ideal humanity, where self-awareness serves as the foundation for creating new artistic ideal states. The artist’s understanding and affirmation of “self” are accomplished through sublime beauty stimulating one’s potential, which is then achieved through learning from exemplary artists. First, the “self state” is an active exploration of personal potential, a long-term accumulation in the practical artistic process, and an artistic display of the individual’s ideal humanity. Wang Guowei emphasized, “the poet beholds a natural scene from a personal perspective, coating everything he sees with a subjective color.” This “color” includes the emotional expression of “joy, anger, sorrow, and happiness” (Wang, 1998, pp. 1–2). This highlights the artist’s self-awareness, observations, feelings, and expressions, culminating in a sublime artistic conception that reflects the personal essence of their mind. The awareness and affirmation of “self” are unique connections and creations between the individual, cosmos, and sublime existence. The artist’s unique style embodies a transformation from “self-ignorance” to “self-awareness,” achieved through a long-term understanding of one’s nature and destiny. Every artist who strives to capture the sublime beauty of their own goals undergoes a transformation from “self-confusion” to “self-awakening,” a process achieved through the long-term understanding and self-expectation of his own nature and destiny. Burke (1990, pp. 126-128) asserts that the factors eliciting the feeling of sublimity encompass not only the stroke itself and elements of surprise but also expectation. This expectation can create a certain tension, thereby initiating a “succession” of self-consciously seeking sublime inspiration, a phenomenon Burke refers to as “the artificial infinite.” This psychological reaction that evokes sublimity reaffirms the interplay between “potential-process-goal,” rooted in the universal systematicity of the human mind. The contemporary value of the “self state” is evident in its ability to inspire the intercultural other to discover their own potential and achieve sublime experiences, often culminating in the development of their own artistic style. For instance, after studying Chinese brush techniques, American abstract artist Mark Tobey expressed that his view of the world had shifted: the trees in front of his Seattle studio were no longer solid objects, but had broken down into smaller solid states, and he stated “the Great Dragon is breathing sky, thunder, and shadow; wisdom and spirit vitalized.” (Tobey, 1951) Therefore, his creative mind was awakened, and through this Tobey went on to develop his own abstract style. This cross-cultural description of the sublime experience carries with it a certain feeling of awe and even fear. Modern psychology experiments have shown that the relationship between the sublime and fear is very complex (Hur et al., 2020). Specifically, compared with the joyful appreciation emotion, fear can significantly increase a person’s experience of the sublime (Eskine et al., 2012).

**Figure 10:**
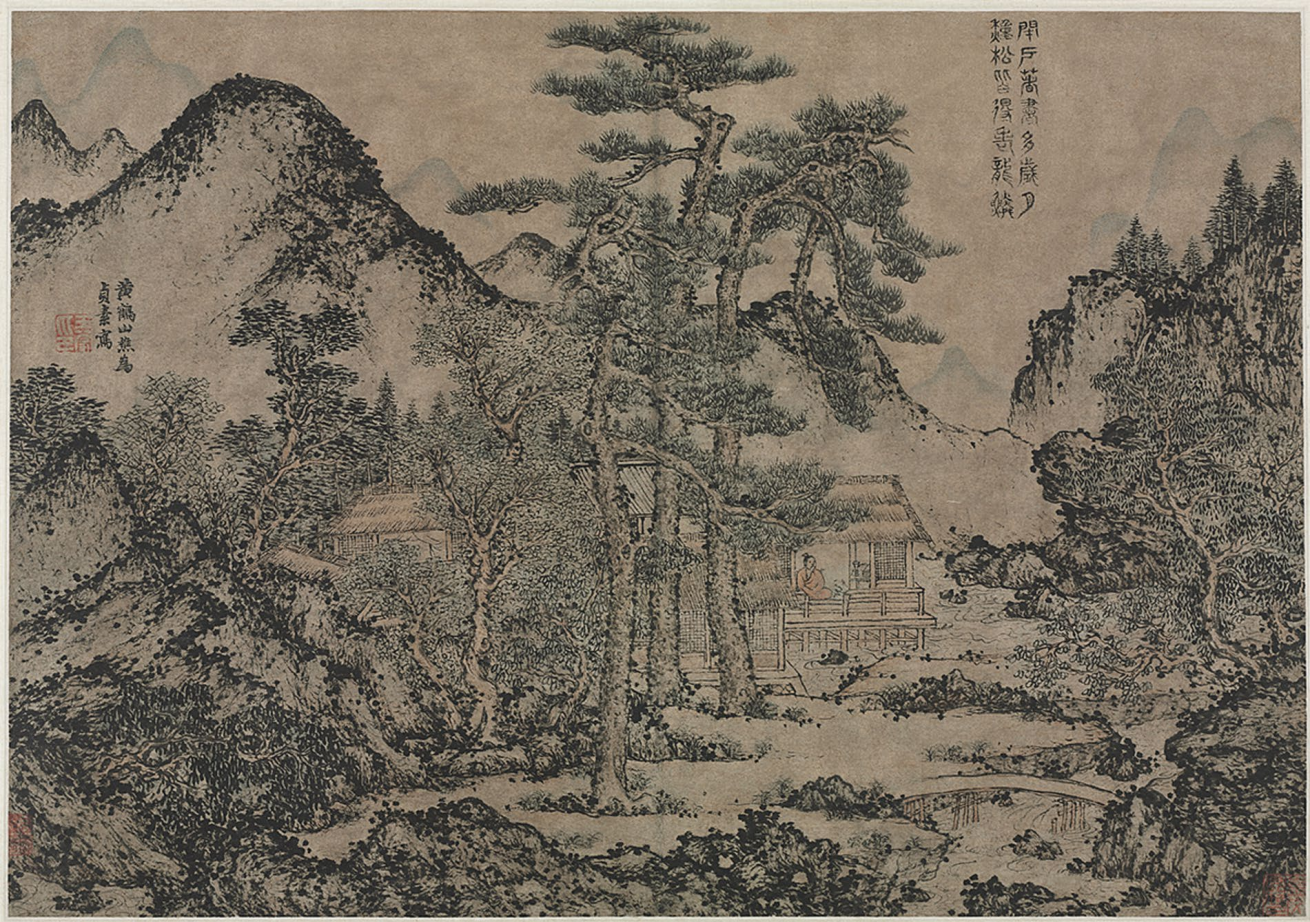
Writing Books under the Pine Trees. Wang Meng. The Cleveland Museum of Art (https://www.clevelandart.org/art/1997.94).

Second, this “self state” arises from one’s awareness of the boundary between “self” and “other.” In the initial stages of copying and learning art, the artist must first abandon their own “self” and enter the aesthetic experience and formal state of the “other.” This allows the artist to perceive and experience the creation of sublime beauty, thus stimulating their own creative potential and style. Through the interaction between the “self” and “other,” the artist gradually refines their unique connection with sublime beauty and ultimately creates the “self state.” A similar experience comes from Longinus (1995, p. 211), who also mentioned in praising Plato: through “imitation of the great prose writers and poets of the past.” For example, in Wang Meng (王蒙)’s *Writing Books under the Pine Trees* (松下著書圖; see Figure 10), ancient pines and majestic mountains create a world that conveys a profound sense of space. Central to the scene is a scholar in a small house, absorbed in his work and indifferent to worldly pursuits. He is one with the beauty of heaven and earth, symbolizing the ideal humanity of the artist. The numerous dots forming the shape of the mountains and trees, a common technique used to depict moss in Chinese paintings, symbolize the vigorous beauty of nature’s life force. While dispersing the viewer’s gaze, they simultaneously evoke a sense of immersion in a tranquil and pure state, as though approaching the depicted scholar’s ideal of transcending worldly pursuits. The experiment showed that there was no significant difference between art and natural stimulation in their ability to evoke sublime experiences. However, compared with natural experiences, art forms can provide “an increased feeling of existential safety” (Chirico et al., 2021).

### 4.2 The “no-self state”

The “no-self state” (無我之境, *wu wo zhi jing*) is achieved through critical thinking, transcending the limitations of the self, and entering a sublime state of the mind. In the process of approaching sublime beauty, once an individual has reached the “self state,” they may become overly attached to individualism. Therefore, one must use critical thought to surpass personal emotions and desires, break free from the constraints of pragmatism, and enter a state of unity with heaven and earth, known as the “no-self state.” The concept of “no-self” originates from Daoist ideals of sublime beauty. Laozi, for example, sought to return to a state of pure and tranquil inaction, akin to the simplicity of an infant’s mind. In terms of sublime spiritual experiences, Zhuangzi’s teachings regarding “losing the self” and “forgetting the self,” as well as the famous story of “Zhuangzi dreaming of himself as a butterfly,” all emphasize the merging of the subject and the object of cognition, integrating the self with the environment and the world, and therefore achieving great freedom (Xu, 2005, p. 105). In the “no-self state,” the individual forgets one’s goals, processes, methods, effects, and meaning in terms of cognition, no longer needing to control their conscious awareness. It is in this void of pleasurable existence that the artist uniquely connects with the sublime beauty of the world.

**Figure 11:**
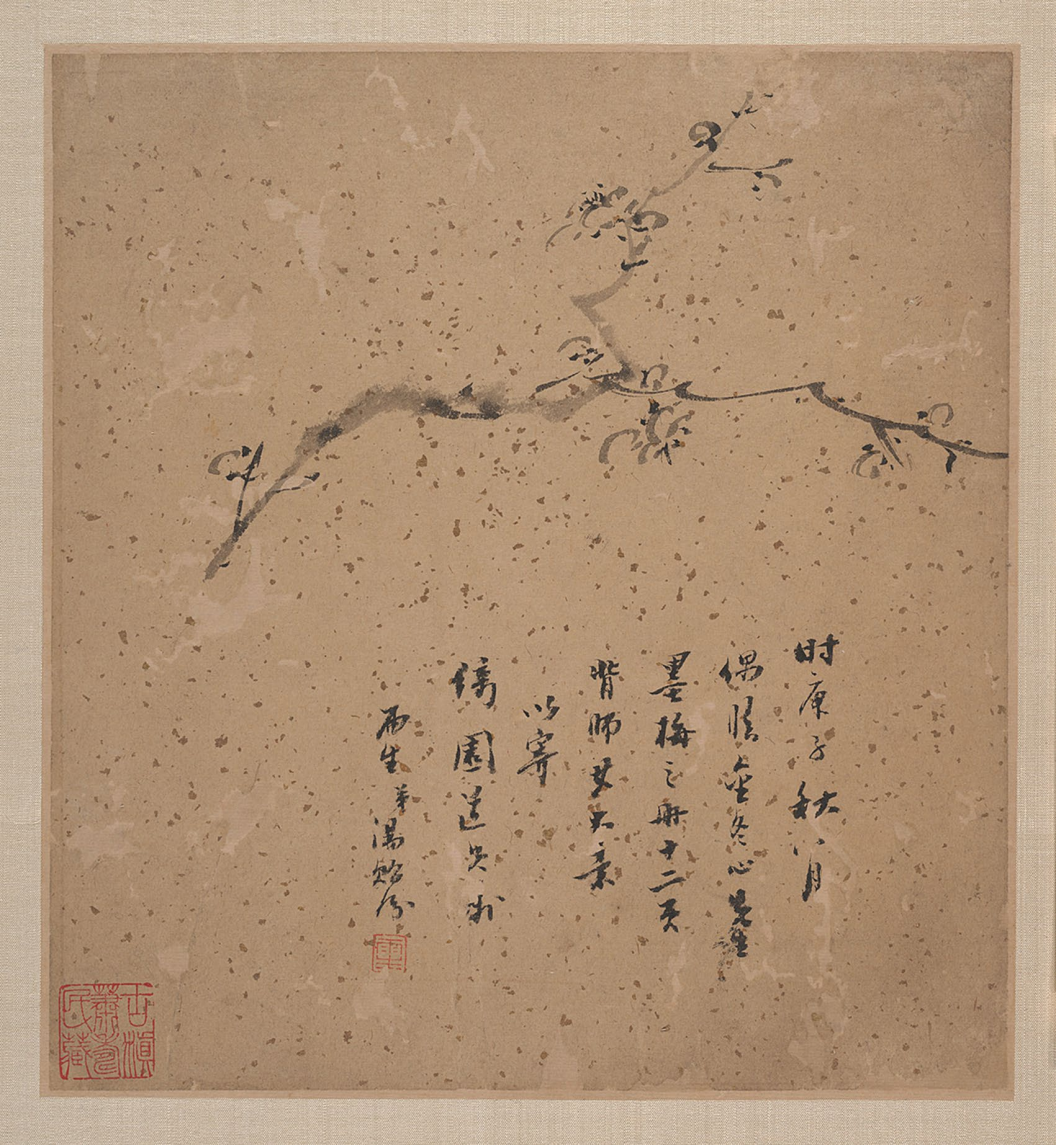
Blossoming Plum (detail). Tang Yifen. The Metropolitan Museum of Art (https://www.metmuseum.org/art/collection/search/72711).

How can one achieve the “no-self state”? In addition to the foundational practice of “focused sincerity” that has been discussed previously, Zhuangzi’s philosophy centers on the concept of “three types of listening,” with an emphasis on “listening with *qi* (以氣聽).” The first is listening with the ears, which is concerned with sensory cognition. The second is to listen with the heart, which emphasizes rational cognition. However, Zhuangzi argues that these two forms of listening, which are primarily focused on objective reality, tend to overload the mind with excessive thoughts, thus creating psychological burdens. Hence, Zhuangzi advocates for a third form of listening—“listening with *qi*.” This kind of “listening” is equivalent to the aforementioned “enlightenment” (*wu*): emphasizing transcending the distinction between subject and object, stopping all sensory and rational cognition, and accepting everything in an open and free state. Therefore, only by clearing the subjective consciousness of the self and breaking the boundary between the self and the object can it be “empty” and have extra space to accept new things. Just like breathing, exhaling can inhale, and thus naturally enter the ethereal state of mind in the process of exhaling and inhaling (Guo,1961, p. 147; Fromm et al., 1960, pp. 115–116). Just like John Cage’s “4′33,” only by stopping playing can you listen to the more subtle voices of the audience and the world. As mentioned earlier, “*q*i” is the connecting force between the individual self and heaven and earth. Because of its nature of being “empty, soft, and yielding (虛柔任物; Guo, 1961, p. 147),” it can encompass things and relax the body and mind. In the process of natural breathing, “*qi*” helps to quiet the mind, allowing the individual to listen to their mind and even to the “sounds” of all things (Maslow, 1971, pp. 119–120). In this state of “meditation (靜坐),” free from any cognitive distinctions between the self and other, one can achieve heightened perception through interoception, reaching a state of clarity and emptiness—an experience of “no-self” (Gao et al., 2019). An example of this is the painting *Blossoming Plum* (墨梅; see Figure 11) by Tang Yifen (湯貽汾). The painting depicts a single plum blossom, surrounded by ample blank space, accompanied by text that brings joy to the viewer. The varied use of ink in the plum blossoms, its gradation from lightness to darkness, and the rhythmic quality of the brushstrokes—quick, slow, and flowing—follow the natural flow of heaven and earth, like a breath of life itself. Through “*qi*,” the artist listens to the growth of all things, creating the “no-self state.”

### 4.3 The “empty state”

The “empty state (空空之境, *kong kong zhi jing*)” represents a sublime ideal state that completely transcends the self and is characterized by the absence of self, language, cognition, and even consciousness. The Chinese word “空 (emptiness)” signifies negation, while “空空 (emptiness of emptiness)” further amplifies this negation—essentially a critique of critical thinking or a negation of negation. Conceptually, “emptiness” (śūnya) means that all things are illusory and unreal, arising from the combination of causes and conditions. The “empty state” signifies a condition in which individuals can transcend mental concepts, including “emptiness,” and truly enter a state of absolute freedom without constraints. This idea originates from Buddhist thought: all phenomena—including the “self”—are empty of intrinsic existence (Van Gordon et al., 2017). This sublime transcendence is also echoed by Western philosophers; for example, Plato’s “absolute beauty” (Cooper, 1997, p. 493), Augustine’s God, who is beyond description (Augustine, 2008, p. 6), and Hegel’s concept of the transcendental sublime entity (Hegel, 1975, p. 363). Combining the viewpoints of Buddhism and psychology, the self is merely an illusion of self-consciousness, which is described as “the sense of self as a set of impersonal mental and physical phenomena, whose interaction creates the illusion of self-consciousness” (Loy, 1992). If we cling to these appearances, we risk misleading our cognition, inducing desires, and generating delusions. Therefore, we must employ negative thinking to see through the transient and perishable beauty of appearances, which will lead us to the truth of our minds, help us distance ourselves from delusions, and ultimately achieve true freedom in personal life. In summary, the distinctions among the three realms discussed in this article can be illustrated using Fromm’s example. The “self state” emphasizes the psychological subject’s cognition, as exemplified by the statement “I see a rose.” The “no-self state” seeks to attain a perspective of “I see it as it or he is,” encapsulated in the phrase “a rose is a rose is a rose” (Fromm et al., 1960, pp. 116–117). In contrast, the “empty state” transcends even the concept of “rose” and the limitations of language itself: “…” To be precise, there should not even be any “…” in the “empty state.” However, in order to convey the concept of “emptiness,” we can only temporarily resort to this omitted and negative expression. In short, the essence of “emptiness of emptiness” lies not only in negating the “no-self,” but also in negating concepts such as “nothingness,” “emptiness,” “mind,” “beauty,” and the “sublime” to approach the Buddhist ideal of the “no-form state (無相狀態),” which Zhuangzi describes as “no-no (無無).” In Buddhist philosophy, all cognition is seen as a product of harmony between causes and conditions, and thus are all an illusory appearance. Therefore, it is only by criticizing the critique and negating negation that one can transcend the fundamental causes of cognitive confusion and attain the ultimate “sublime beauty,” thereby achieving a stable state of nirvana free from delusion.

**Figure 12:**
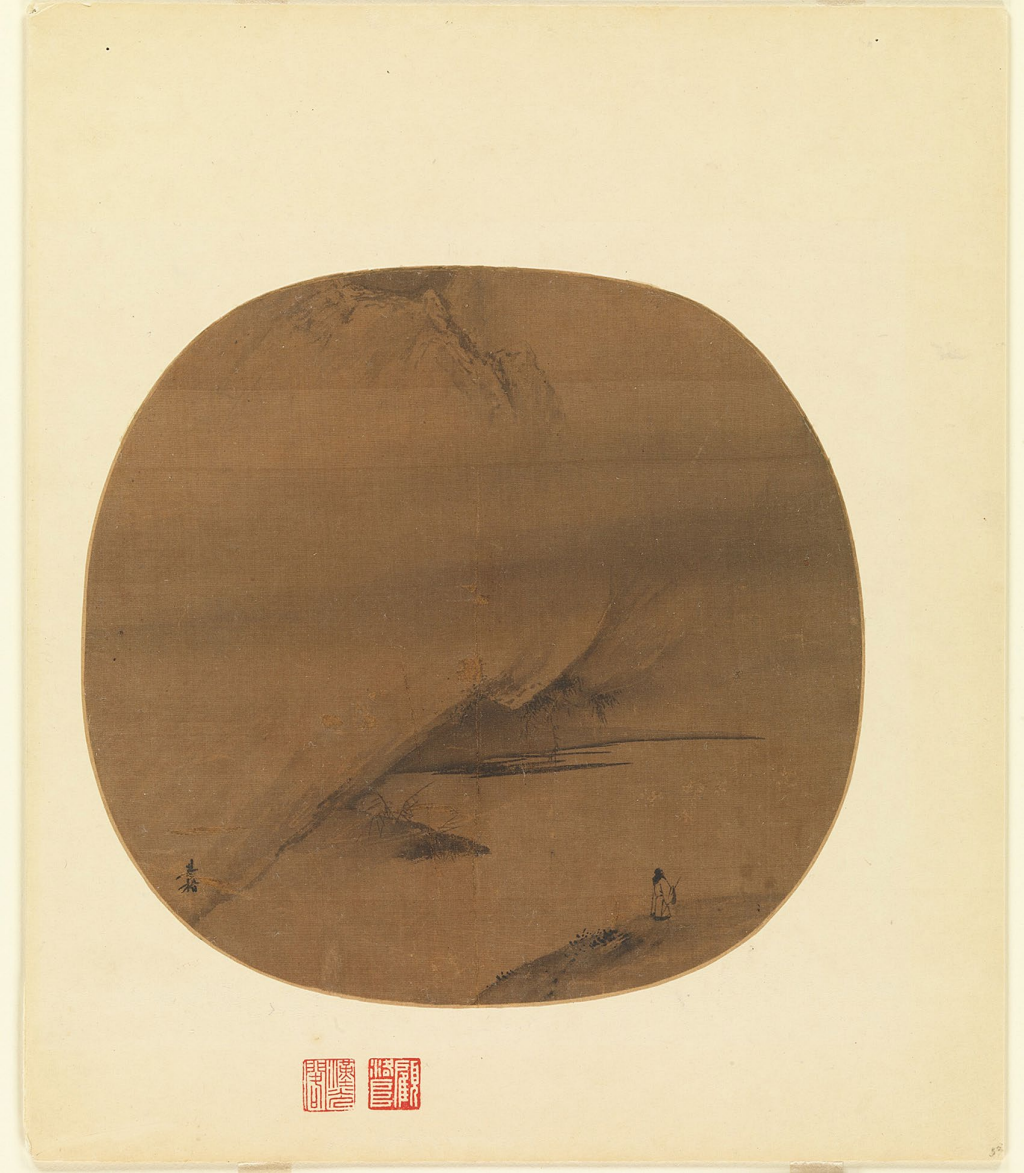
Poet Strolling by a Marshy Bank. Liang Kai. Metropolitan Museum of Art (https://www.metmuseum.org/art/collection/search/40090).

At this point, Chinese art transcends the concrete imagery and expression of brushstrokes, aiming instead to explore infinite possibilities beyond the limits of language. Language and art, in this context, are mere tools for facilitating initial understanding; however, ultimately, they must be negated to realize the cognition of sublime beauty. In other words, the ultimate goal is to transcend the state of “nothingness” and enter the “almost indescribably sublime” of “neither perception nor nonperception” (Keown, 2013, pp. 35–36). For example, the Buddhist concept of the beauty of nirvana transcends individual perceptions of reality and cannot be described using language. Despite this, Chinese painting still uses symbolic forms to express the “empty state,” providing a medium through which to approach and understand sublime beauty. An example of this is found in Chen Hongshou (陳洪綬)’s *No Words to Speak* (無法可說), which features an image of a Buddhist monk facing a kneeling devotee, neither speaking. The silence between them signifies that not speaking is itself a form of Dharma. Another example is Liang Kai (梁楷)’s *Poet Strolling by a Marshy Bank* (澤畔行吟圖; see Figure 12), in which a small figure stands before a vast and empty universe, imparting a sense of existential reflection and psychological pressure to the viewer. To further illustrate the meaning of “emptiness,” some extreme approaches include works where the painter Crazy Gan (甘疯子) produces calligraphy or simple brushstrokes, and once the audience has viewed the piece, the artwork is destroyed (Deng, 1964, p. 55). The reasoning behind this approach is that “beauty” is finite, and when expressing the sublime state, the limitations of this “cognitive” experience must constantly be negated, lest it become an obstacle to entering ultimate sublime beauty. Thus, it is through this process of negating negation and transcending transcendence that one may enter the “empty state.”

In conclusion, the discussion presented above, grounded in the “potential-process-goal” research model, reveals three relationships between the mind and sublime beauty within the goal dimension of Chinese art: the “self state,” “no-self state,” and “empty state.” The latter two realms, in particular, may benefit from greater engagement by researchers and readers, who can explore these dimensions through varied approaches, so as to discover the suppressed potential and maintain the interactive state between mind and sublime beauty in daily life and creative process (see Table 3).

**Table 3:** Summary of key arguments of relationships with the dimension of goals.

The sublime goals	The thoughts of recognize sublime beauty	The ways to achieve sublime beauty
The “self state”	Refers to the conscious pursuit and expression of one’s ideal humanity, where self-awareness serves as the foundation for creating new artistic ideal states	The unique connection and creation between the individual and the universe and the sublime beauty, showing the unique artistic style of the self
The “no-self state”	Achieved through critical thinking, transcending the limitations of the self, and entering a sublime state of the mind	One can achieve heightened perception through interoception, reaching a state of clarity and emptiness
The “empty state”	Represents a sublime ideal state that completely transcends the self and is characterized by the absence of self, language, cognition, and even consciousness	Through the negation of negation, we can transcend the temporary and fleeting beauty of the appearances seen by the naked eye, thus staying away from delusions and ultimately achieving the true awakening of one’s sublime life

## 5 Conclusion

First, human potential accommodates both the uniqueness of the individual and the universality of the sublime. This dual focus allows individuals to fully explore and realize their ideal humanity during the process of self-actualization. This perspective not only helps modern individuals enhance their self-awareness and confidence but also inspires people from diverse cultures to tap into their inner sublime ideal humanity through Chinese cultural practices.

Second, in artistic practice, a mental state of focused sincerity is emphasized as a means of unlocking inspiration. Creative thinking combines the “method of equilibrium” of emotional regulation with the “*qi*” of personal cultivation to achieve the concept of the “method of no-method.” The method of recognizing the sublime involves the gradual accumulation of embodied experience of everyday life (“gradual enlightenment”) and cultivation of innate talents through sudden, key moments of insight (“sudden enlightenment”).

Third, the artistic goal of ideal humanity is a “creative concept” that integrates innate potential, cognitive experience, and creative inspiration. This process is divided into three levels based on the relationship between the self and the sublime: (i) “the self state,” which focuses on the relationship between the individual and sublime existence; (ii) “the no-self state,” which transcends the limitations of the self; and (iii) “the empty state,” where cognition undergoes the negating of negation, allowing for the realization of the “empty state.”

Fourth, in the research model of “potential-process-goal,” the relationship between ideal humanity and sublime beauty in Chinese art is shaped by the constant interaction and mutual influence between potential, process, and goals as the unique research model. This interaction forms a cyclical, open, and flowing relationship. Only by employing this research model, which respects the complexities of the mind, can we uncover deeper insights into the intricate relationship between the ideal humanity and sublime beauty. Future research should continue to build upon this model, incorporating cross-cultural and interdisciplinary perspectives to elucidate a more nuanced and diverse interaction between the mind and sublime beauty, thereby exploring additional possibilities for the “*sheng-sheng*” development of human beings.

## Data Availability

The original contributions presented in the study are included in the article/supplementary material, further inquiries can be directed to the corresponding authors.
